# Comparison of Postoperative Analgesic Effects Between Nalbuphine and Fentanyl in Children Undergoing Adenotonsillectomy: A Prospective, Randomized, Double-Blind, Multicenter Study

**DOI:** 10.3389/fphar.2020.597550

**Published:** 2020-12-09

**Authors:** Fang Chen, Cheng-Yu Wang, Jianmin Zhang, Fang Wang, Mazhong Zhang, Hongbin Gu, Xingrong Song, Jia Chen, Yang Li, Yu-Hang Cai, Jun Li, Qing-Quan Lian, Junzheng Wu, Hua-Cheng Liu

**Affiliations:** ^1^ Key Laboratory of Anesthesiology of Zhejiang Province, Department of Anesthesiology, Perioperative and Pain Medicine, The Second Affiliated Hospital and Yuying Children's Hospital of Wenzhou Medical University, Zhejiang, China; ^2^ Department of Anesthesiology, Beijing Children’s Hospital, Capital Medical University, Beijing, China; ^3^ Department of Anesthesiology, Shanghai Children’s Medical Center, Shanghai, China; ^4^ Department of Anesthesiology, Guangzhou Women and Children’s Medical Center, Guangzhou, China; ^5^ Department of Anesthesiology, Cincinnati Children’s Hospital, Cincinnati, OH, United States

**Keywords:** nalbuphine, fentanyl, analgesic, adenotonsillectomy, children

## Abstract

**Objective:** There is no universal agreement on optimal pharmacological regimens for pain management during surgeries. The aim of this study to compare the postoperative analgesic effects of nalbuphine with fentanyl in children undergoing adenotonsillectomy.

**Design, Setting, Participants:** We conducted a prospective, randomized, double-blind, non-inferiority and multicenter trial in 311 patients admitted to four different medical facilities in China from October 2017 to November 2018.

**Main Outcome Measure:** The primary outcome was postoperative pain score. The secondary outcomes were as follows: the numbers of patients who developed moderate or severe pain (FLACC ≥4 points); time to first rescue analgesic top up and the actual number of rescue pain medicine given in pain control in post-anesthesia care unit (PACU), and additional analgesics requirement (received ≥2 rescue analgesics or/and other analgesics except study medications administered in PACU and ward); emergence and extubation time; Waking up time; time of PACU stay, and other side effects (desaturation, nausea/vomiting etc.).

**Results:** A total of 356 children were screened and 322 patients were randomized. The mean age was 5.8 (5.5, 6.1) in the nalbuphine group and 5.6 (5.3, 5.8) in the fentanyl group (*p* = 0.2132). FLACC score of nalbuphine group was lower than that of fentanyl group upon patients' arrival at PACU (*p* < 0.05). The time to first required rescue dose of pain drug for nalbuphine group was longer than for the fentanyl group (2.5 vs 1.2 h, *p* < 0.0001). Only one patient (0.6%) in nalbuphine group presented a slow respiratory rate (RR) at 9/min while 29 patients (18.5%) in fentanyl group developed slow RR ≤10/min in PACU. Meanwhile, SpO_2_ was lower in the fentanyl group at 10 min after patients’ arrival in PACU (*p* < 0.05). The other profiles observed from these two drug groups were similar.

**Conclusion:** Nalbuphine provided better pain relief with minimal respiration depression than fentanyl in children undergoing Adenotonsillectomy.

## Introduction

Adenotonsillectomy or tonsillectomy is the most commonly performed procedure for the treatment of obstructive sleep disorder, especially in the pediatric population (Reckley et al., 2018). Upper airway obstruction is the most frequent and serious postoperative complication following this procedure (Isaiah et al., 2018). There are plenty of attributes to this situation, including the tissue swelling, hemorrhage and the side effects of general anesthesia. It is also noteworthy that large dose of opioids may suppress respiration and precipitate airway obstruction after surgery (Overdyk and Hillman, 2011), but suboptimal dose of opioids may has left patients restless and agitated due to poor pain control in post-anesthesia care unit (PACU).

There is no universal agreement on attenuating reflex responses to nociceptive stimuli during tonsillo-adenoidectomy. However, the common practice is to administer opioids or non-steroidal anti-inflammatory agents (NSAID), alone or in combination. The extensive studies concerning the pharmaceutical management of postoperative pain and pain-related restlessness in the recovery room can be found throughout the literature. Opioids and/or NSAIDs may be administered as premedication (Atkinson et al., 1987; Bone and Fell, 1988; Nordbladh et al., 1991; Rusy et al., 1995), during induction of anesthesia (Watters et al., 1988; Thiagarajan et al., 1993), in the middle or at the end of surgery (Nordbladh et al., 1991; Gunter et al., 1995; Sutters et al., 1995).

Nalbuphine, an active agonist–antagonist opioid on mu receptors in the medulla and on kappa receptors in the cerebral cortex (Martinelli et al., 2014) has been demonstrated to provide effective analgesic (Bahar et al., 1985). It has been well-known that its analgesic potency is equivalent to that of morphine and its onset time is similar to fentanyl (Beaver and Feise, 1978; Dick et al., 1992; Zeng et al., 2015). Also, nalbuphine possesses a ceiling effect regarding respiratory depression at 0.2–0.4 mg kg^−1^ (Gal et al., 1982; Bahar et al., 1985) cardiovascular stability and rapid recovery to wakefulness (Lee et al., 1981; Zsigmond et al., 1987). These properties may make nalbuphine a more ideal and safer analgesic for children undergoing adenotonsillectomy.

Fentanyl, a potent, short-acting agonist opioid, has been widely used as one of the main anesthetic agents during cardiac surgeries and as an important component in balanced anesthesia during general surgeries (Stanley and Webster, 1978; Bovill et al., 1984; Flacke et al., 1985; Pandit et al., 1987). However, fentanyl is associated with an increased risk of respiratory depression at the end of surgery (Adams and Pybus, 1978). Nalbuphine has the same effect as morphine for analgesia (Beaver and Feise, 1978), but has barely been studied in children undergoing adenotonsillectomy in which fentanyl is the main choice of the drug for perioperative pain management (Vittinghoff et al., 2018).

The aim of this study was to compare the analgesic effectiveness and the impact on respiration between nalbuphine and fentanyl in children undergoing adenotonsillectomy.

## Methods

### Study Design

This was a prospective, randomized, double-blind, non-inferiority multicenter study, which were carried out together by four different medical facilities led by the Second Affiliated Hospital and Yuying Children’s Hospital, Wenzhou Medical University of China from October 2017 to November 2018. This clinical trial was approved by institutional review board prior to start (Reference No. 2015-09) and was registered at www.chictr.org.cn on October 13, 2017 (ChiCTR-IPR-17012969). A written informed consent for participants was provided by the parents or a legal guardian.

### Study Population

A total of four hundred children, scheduled for combined Tonsillectomy and Adenoidectomy under general anesthesia were enrolled initially. Inclusion criteria were as follows: Aged 3–10 years old male or female; Body mass index (BMI) ≥15.5 kg/m^2^ and ≤24.5 kg/m^2^ and body weight above 12 kg; American Society of Anesthesiologists physical status (ASA) Class I or II. Exclusion criteria are as follows: Allergy to nalbuphine or its components; preoperative use of any analgesics, sedatives, anti-emetics or antipruritic 72 h before surgery; running fever with body temperature higher than 38°C 24 h before surgery or display of the symptoms of acute upper respiratory tract infection and bronchial asthma; severe obstructive sleep apnea syndrome and arrhythmia.

### Data Collection

A case report form (CRF) was designed for the registration of clinical data and study results. Data were stored in a password-protected computer for the concealment of patients’ confidentiality. The guideline of good clinical practice (GCP) was closely followed during the study. One investigator was specifically assigned to the job for data collection, filing, and transfer and another one to verify the data’s accuracy and safety.

### Randomization and Blindness

Eligible recruits were randomly assigned into either nalbuphine or fentanyl group in a 1:1 ratio by a computer-generated digit-number program (SAS PLAN; SAS Institute Inc.) in each individual participating medical center. An assignment number was sealed in an envelope and was revealed just before the drug’s administration.

The medications were prepared according to the group allocation before the surgery started. For the intraoperative dosing, nalbuphine (100 µg·kg^−1^) or fentanyl (1 µg·kg^−1^) (Yichang Humanwell Pharmaceutical CO) was diluted up to 10 ml in a syringe with isotonic saline. For postoperative rescue dosing, nalbuphine (50 µg·kg^−1^) or fentanyl (0.5 µg·kg^−1^) was made up to 5 ml in a syringe with normal saline.

Each syringe was labeled with an individual assignment number, which matched a pre-sealed envelope. The anesthesiologist who administered the injections, the outcomes evaluator, the parents or guardian and the children were blinded to the allocation of study drugs.

### Clinical Protocol

Patients’ preoperative fasting status was confirmed to meet the ASA guideline and a peripheral venous access was established prior to surgery. No premedication was used according to the study protocol. Noninvasive blood pressure (NIBP), heart rate (HR), and oxygen saturation (SpO_2_) were continually monitored 30 min before surgery. Anesthesia was induced with propofol 2.5 mg kg^−1^ and remifentanil 2.5 µg·kg^−1^ intravenously. Endotracheal intubation was facilitated with cisatracurium 0.6 mg kg^−1^. Mechanical ventilation was carried out with a 1:1 mixture of nitrous oxide and oxygen at a total flow of 2 L/min.

Anesthesia was maintained with propofol 3–15 mg kg^−1^·h^−1^ and remifentanil with 0.1–0.5 µg·kg^−1^·min^−1^. Heart rate and mean arterial blood pressure were kept within 80–120% of baseline values. The end-expiratory carbon dioxide partial pressure (PETCO_2_) was maintained around 35–45 mmHg by adjusting the respiratory rate (RR) and tidal volume. Repetitive cisatracurium injection was given as needed. Intravenous infusion of propofol and remifentanil were discontinued right away after tonsillar and adenoid were removed. At the same time, nalbuphine 100 μg/kg or fentanyl 1 μg/kg, in 10 ml normal saline was slowly injected intravenously over 1 min. Ondansetron (0.1 mg/kg, max 4 mg) was given and patents were reversed with neostigmine 0.02 mg/kg and atropine 0.01 mg/kg, and then, were allowed to emerge from anesthesia. A wake-extubation technique was performed in the operating room. Children were sent to PACU and were observed for at least 1 h before the discharge to ward after having met a modified Aldrete score 9 (Aldrete and Kroulik, 1970). The postoperative pain was scored with FLACC (Merkel et al., 2002) (Table 1).

**TABLE 1:** Face legs activity consolabilty and cry (FLACC) scale.

Categories	Scoring		
	0	1	2
Faces	No paticular expression	Occasional grimace or	Frequent to constant
	Or smile	Frown, withdrawn,	Quivering chin,
	—	disinterested	Clenched jaw
Legs	Normal position or	Uneasy, restless, tense	Kicking or legs drawn
	Relaxed	—	Up
Activity	Lying quietly, normal	Squirming, shifting	Arched, rigid or
	Position, moves easily	Back and forth, tense	Jerking
Cry	No cry (awake or asleep)	Moans or whimpers	Crying steadily,
	—	occasional complaint	Screams or sobs,
	—	—	Frequent complaints
Consolability	Content, relaxed	Reassured by	Difficult to console
	—	occasional touching	or comfort
	—	Hugging or being talked	
	—	To distractible	—

**FIGURE 1:**
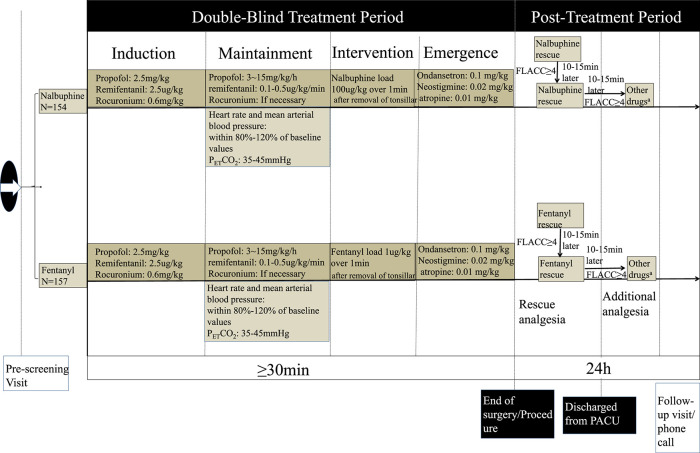
Study schema.

For children who suffered moderate or severe pain (FALCC ≥4 points) in PACU, a pain rescue drug, either nalbuphine or fentanyl in line with intraoperative group allocation, was given intravenously. Then, FLACC score was assessed again 10–15 min later. If the score remained ≥4 points, the second dose of rescue analgesic was administered. After that, the decision to give additional analgesic (either narcotics or NSAIDS) based on the reevaluation of FLACC score, and was totally at the discretion of anesthesiologist who was in charge of patient care. The recorded drug dose and frequency of drug administration was shown in Figure 1.

### Outcomes

The primary outcome was postoperative pain score. All children were assessed by FLACC at the following time point: 0, 10, 20, 30, 45 min, 1, 2, 6, 12 and 24 h after PACU admission. The secondary outcomes were as follows: the number of cases with moderate or severe pain (FLACC ≥4 points); the number of rescue pain medication administered in PACU and ward; time of emergence and extubation; time of recovery; vital signs (blood pressure, heart rate, RR, oxygen saturation) in PACU; parent’s overall satisfaction with analgesia over 24 h postoperatively.

### Statistical Analysis and Definitions

Based on the preliminary study data of 30 children from the Second Affiliated Hospital and Yuying Children’s Hospital of Wenzhou Medical University, the effect size of non-inferiority analgesia between the nalbuphine group and the fentanyl group was 1.02. A sample size of 296 children (149 children per group) provided 90% power at a two-sided *α* of 0.05 to detect the difference between the two groups. We planned to have 356 enrollments (178 per group) to allow for up to 20% dropout of children who failed to complete the study.

The anesthesia emergence and extubation time meant the time span from the end of the surgery to the extubation. The waking up time was the time span from the moment to discontinue anesthetics to the point when child was fully conscious.

Statistics analysis was conducted by SPSS version 24.0 for windows (SPSS lnc., Chicago, IL, United States). The normality of distribution of continuous variables was tested by one-sample Kolmogorov-Smirnov test. The continuous variables of normal distribution were expressed as mean (SD); non-normal variables were reported as median (interquartile range [IQR]). Means of two continuous normal distributed variables were compared by independent samples student’s test. Mann-Whitney U test and Kruskal-Wallis test were used to compare two groups of non-normally distributed variables respectively. Data were expressed in the number and percentage of categorical variables. The frequencies of categorical variables were compared with Pearson χ^2^ or Fisher’s exact test whenever it was appropriate. The preoperative pre-dosing baseline data was compared with post-dosing data by ANOVA test through repeated measures. Only when ANOVA test was significant, the *p* value for pairwise comparisons was computed with Student’s test with Bonferroni correction. All tests were two-sided, and *p* < 0.05 was considered to be statistically significant.

## Results

**FIGURE 2:**
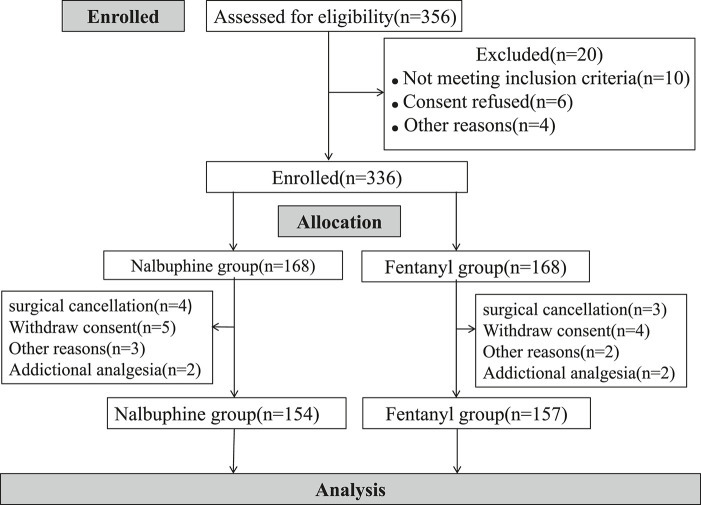
Patient disposition.

Three hundred fifty six children were initially assessed for eligibility and 20 were excluded prior to randomization because of unsatisfied inclusion requirement or refusing to participate. A total of 336 children was evenly and randomly assigned to each group. 14 children in nalbuphine group and 11 in Fentanyl group were removed from the study later for following reasons: additional analgesia having either received additional opioids or other pain medicine beyond the study protocol; withdrawal consent, surgical cancellation and etc. In the end, 154 patients were treated with nalbuphine and 157 with fentanyl (Figure 2). Demographic and clinical data were summarized in Table 2. The median age of participants was 5.2 years (IQR, 4.3–6.8 years) and the median weight of participants was 20.0 kg (IQR, 11.5–27.3 kg). There were no differences in children characteristics between nalbuphine and fentanyl group.

**TABLE 2:** Demographic data of patients.

	Overall (N = 311)	Nalbuphine (n = 154)	Fentanyl (n = 157)	*p*-value
Sex (Male/Female)	220/91	107/47	113/44	0.7198
Age (years)	5.7 (5.5, 5.9)	5.8 (5.5, 6.1)	5.6 (5.3, 5.8)	0.2132
Weight (kg)	22.7 (21.9, 23.6)	23.0 (21.7, 24.2)	22.5 (21.3, 23.6)	0.774
Height (cm)	114.7 (113.0, 116.4)	115.5 (113.2, 117.8)	113.8 (111.3, 116.3)	0.6632
BMI (kg/m^2^)	16.9 (16.6, 17.1)	16.9 (16.5, 17.3)	16.8 (16.4, 17.1)	0.5844
BBB (°C)	36.6 (36.5, 36.6)	36.6 (36.5, 36.6)	36.6 (36.5, 36.6)	0.7519
Types of operation				0.7995
Curettage	53 (17.0)	28 (18.2)	25 (15.9)	
Electrocoagulation	258 (83.0)	126 (81.8)	132 (84.1)	
ASA				0.3539
I	215 (69.1)	104 (67.5)	111 (70.7)	
Ⅱ	96 (30.9)	50 (32.5)	46 (29.3)	

ASA, American Society of Anesthesiologists; BMI, body mass index (calculated as Weight in kilograms divided by height in meters squared); BBB, basal body temperature. Values are number (percentage, %) for number of ASA and types of operation; Other values are mean (95% CI).

### Analgesia

**FIGURE 3:**
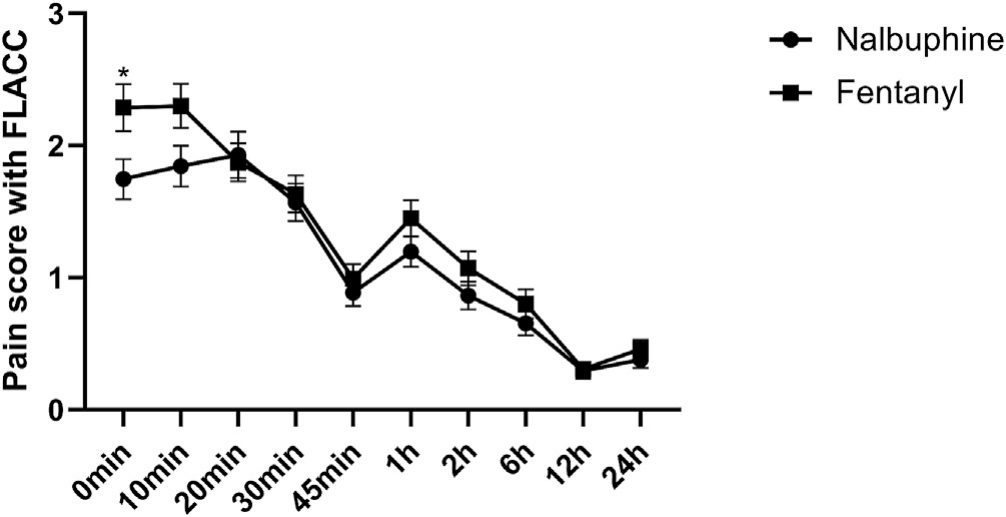
Postoperative FLACC score for pain relief receiving Nalbuphine (blue line) or Fentanyl (red line) (Mean ± SD). The average of FLACC scores among the patients at the arrival of PACU (0 min) was significantly higher in fentanyl group than that in nalbuphine group (*p* = 0.0364), but no differences were found for the rest of the assessment time-points.

Complete sets of FLACC scores were collected in 311 patients. The average of FLACC scores among the patients at the arrival of PACU (0 min) was significantly higher in fentanyl group than that in nalbuphine group, but no differences were found for the rest of the assessment time-points (Figure 3). However, the total number of cases with FLACC ≥4 at 0 min of PACU was not significantly different between two groups. At 1 and 2 h PACU time points, Nalbuphine group had significantly fewer children (10 and 10) with FLACC scores ≥4 than fentanyl group (51 and 26 respectively) (Table 3). The time to first required rescue (The time from arriving PACU to first required dose of pain drug) dose of pain drug in nalbuphine group was longer than for the fentanyl group (Table 4). Six children in the fentanyl group and five in the nalbuphine group required more than two doses of rescue pain drugs and other analgesics. All patients had satisfactory pain management before discharge to ward.

**TABLE 3:** Number of patients at each time point in which the FLACC ≥4 points.

	Overall (N = 311)	Nalbuphine (n = 154)	Fentanyl (n = 157)	*p*-value
PACU	31 (10.0)	15 (9.7)	16 (10.2)	0.9396
PACU (10 min)	35 (11.3)	17 (11.0)	18 (11.5)	0.9354
PACU (20 min)	7 (2.3)	2 (1.3)	5 (3.3)	0.1986
PACU (30 min)	19 (6.2)	7 (4.6)	12 (7.8)	0.3585
PACU (45 min)	14 (5.4)	4 (3.1)	10 (7.8)	0.156
PACU (1 h)	61 (20.1)	10 (6.8)	51 (32.7)	<0.0001
PACU + 2 h	36 (11.7)	10 (6.5)	26 (16.7)	0.0094
PACU + 6 h	15 (4.8)	5 (3.3)	10 (6.4)	0.3137
PACU + 12 h	8 (2.6)	4 (2.6)	4 (2.5)	0.748
PACU + 24 h	11 (3.5)	6 (3.9)	5 (3.2)	0.9652

PACU, post-anesthesia care unit. Value are numbers (percentages, %).

**TABLE 4:** Number of patients at each time point in which the respiratory rate was below 10 breath/min.

	Overall (N = 311)	Nalbuphine (n = 154)	Fentanyl (n = 157)	*p*-value
PACU	10 (3.2)	0 (0.0)	10 (6.4)	0.0017
PACU + 10 min	14 (4.5)	0 (0.0)	14 (8.9)	<0.0001
PACU + 20 min	1 (0.3)	0 (0.0)	1 (0.6)	1
PACU + 30 min	2 (0.6)	0 (0.0)	2 (1.3)	0.4985
PACU + 45 min	2 (0.6)	1 (0.7)	1 (0.6)	0.4895
PACU + 60 min	1 (0.3)	0 (0.0)	1 (0.6)	1
Total	30	1	29	

Value are number (percentage, %).

### Respiration

**FIGURE 4:**
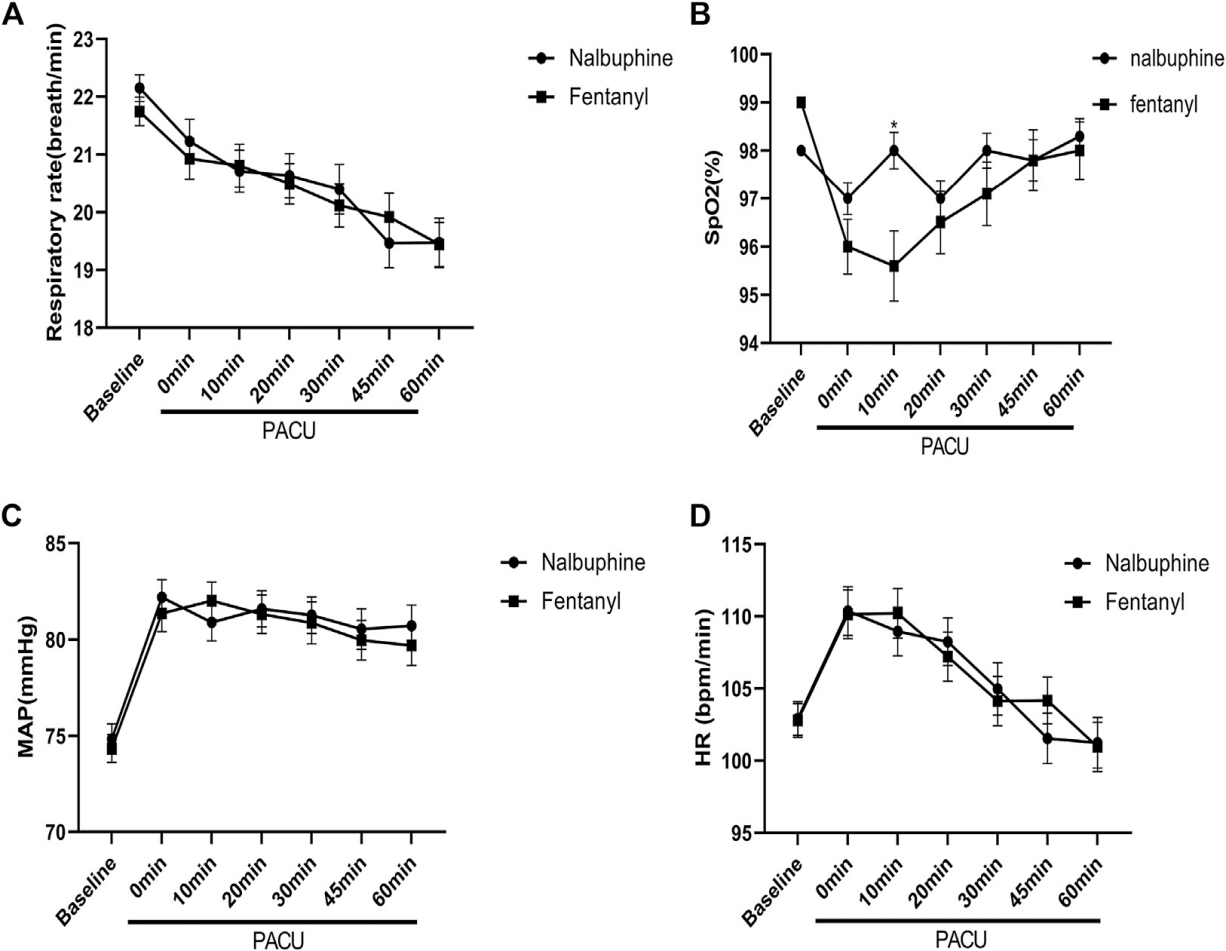
Perioperative feature. **(A):** Respiratory rate (RR) vs. time in patients receiving Nalbuphine or Fentanyl (Mean ± SD). No significant difference between groups. **(B):** SpO_2_ vs. time in patients receiving Nalbuphine or Fentanyl (Mean ± SD). There was marked difference in recorded SP02 levels between the two groups, in which the averaged SpO_2_ was significant lower in Fentanyl group than in nalbuphine group at 10 min after arriving PACU (*p* = 0.0009), but no differences were found for the rest of the assessment time-points. **(C):** Mean arterial pressure (MAP) vs. time in patients receiving Nalbuphine or Fentanyl (Mean ± SD). No significant difference between groups. **D:** Heart rate (HR) vs. time in patients receiving Nalbuphine or Fentanyl (Mean ± SD). No significant difference between groups.

Since patients were mechanically ventilated during the surgery, only post-extubation data were used to assess the effect of analgesics on respiration. Overall, there was no significant difference in postoperative RR between two groups (Figure 4). However, the total number of cases with RR lower than l0 breath/min was significantly fewer in nalbuphine group than in fentanyl group both at the arrival of PACU and 10 min after arrival of PACU (Table 5). Only one patient (0.6%) in nalbuphine group displayed a RR of 9 breath/min at 1 h after entering the PACU, while 29 children (18.5%) in the fentanyl group developed a RR ≤10 breath/min in PACU (Table 5). At 10 min after entering the PACU, 15 patients, all from the fentanyl group, were observed with a RR ≤10 breath/min. Among those patients, 10 experienced episodes of hypoxemia (SpO_2_ <90 lasted for ≥5 s in PACU; eight were rapidly normalized with oxygen supplement through face mask and two were relieved after brief assisted mask ventilation with 100% oxygen. None of the children in the nalbuphine group developed hypoxemia. There was remarked difference in recorded SpO_2_ levels between the two groups, in which the averaged SpO_2_ was significant lower in Fentanyl group than in nalbuphine group at 10 min after arriving PACU (Figure 4).

**TABLE 5:** Extubation time, surgery duration, intraoperative remifentanil consumptions, hemorrhage during operation, time to 1st rescue analgesic and analgesic satisfaction.

	Overall (N = 311)	Nalbuphine (n = 154)	Fentanyl (n = 157)	*p*-value
Extubation time (min)	16.4 (15.5, 17.2)	15.9 (14.8, 17.0)	17.9 (16.7, 19.2)	0.2321
Surgery duration (min)	39.8 (38.4, 41.3)	39.9 (37.8, 41.8)	39.8 (37.8, 41.8)	0.9878
Remifentanil (μg)	308. 1 (291.4, 324.7)	310 (285.4, 336.2)	330.1 (305.3, 355.0)	0.6953
Hemorrhage (mL)	32.0 (30.1, 33.9)	33.1 (30.1, 36.0)	26.2 (24.2, 28.2)	0.8736
Time to 1st rescue analgesic (hours)	3.4 (3.2, 3.6)	2.5 (2.3, 2.7)	1.2 (1.1, 1.3)	<0.0001
Analgesic satisfaction				0.285
1	151 (48.6)	84 (54.5)	67 (42.7)	
2	140 (45.0)	66 (42.9)	76 (48.4)	
3	19 (6.1)	4 (2.6)	12 (7.6)	
4	1 (0.3)	0 (0.0)	2 (1.3)	
Propofol (mg)	61.2 (59.0, 63.4)	62.0 (58.8, 65.2)	60.2 (57.1, 63.3)	0.4440

Analgesic satisfaction: 1 point, Highly satisfied; 2 point, satisfied; 3 point, dissatisfied; 4 point, Not satisfied at all. Values are mean (95% CI) for extubation time, surgery duration, intraoperative remifentanil consumptions, Propofol, hemorrhage or number (percentage, %) for analgesic satisfaction.

### Perioperative Feature

There were no significant differences found in other comparable parameters, including: the time span of emergence and extubation, surgery duration, intraoperative remifentanil consumptions, time to first rescue analgesic, intraoperative hemorrhage and analgesic satisfaction (Table 4). There were also no statistically significant differences in mean arterial pressure (MAP) and heart rate (HR) between two groups (Figure 4).

### Safety

Delayed recovery, waking time, hypotension, bradycardia, nausea, vomiting, and pruritus were similar between the two groups throughout the study course. (Table 6).

**TABLE 6:** Postoperative adverse events.

	Overall (N = 311)	Nalbuphine (n = 154)	Fentanyl (n = 157)	*p*-value
Delayed recovery	0 (0.0)	0 (0.0)	0 (0.0)	
Respiratory depression	15 (4.9)	0 (0.0)	15 (9.6)	<0.0001
Hypotension	0 (0.0)	0 (0.0)	0 (0.0)	
Bradycardia	0 (0.0)	0 (0.0)	0 (0.0)	
Nausea, vomiting	32 (10.3)	13 (8.4)	19 (12.1)	0.6197
Pruritus	3 (1.0)	0 (0.0)	3 (1.9)	0.119
Awaking time (min)	23.2 (21.3, 25.0)	23.5 (21.6, 25.3)	22.9 (21.0, 24.7)	0.5657
Hypoxemia	10 (3.2)	0 (0)	10 (6.3)	<0.0001

Respiratory depression is defined as RR < 8 breath/min. Awaking time are mean (95% CI). Other Value are number (percentages, %).

## Discussion

This was a prospective, randomized double-blind, multicenter study designed to evaluate the effectiveness of postoperative pain relief and the safety of nalbuphine and fentanyl. Our study results have shown that Nalbuphine, an opioid agonist–antagonist, presented a better pain-relief effect with minimal respiratory depression than fentanyl in children undergoing adenotonsillectomy.

Adenotonsillectomy is one of the most performed surgeries in children, and postoperative pain, such as sore throats and earaches, are common and could persist for up to 2 weeks (Reckley et al., 2018). To date, pharmaceutical approaches remain the mainstream of pain management for adenotonsillectomy and the commonly used medications include opioids, non-steroidal anti-inflammatory drugs (NSAIDS) and local anesthetics etc. Morphine, one of the most classic and consumed opioids, has a strong pain-relief effect, but with high incidence of postoperative nausea and vomiting (PONV), and respiratory depression compared with other forms of analgesics (Zeng et al., 2015). Fentanyl, another potent, short-acting opioid agonist, is also a widely used pain-killer during short surgical procedures and however, the major worry to clinicians is that the utility of fentanyl has been noted to be quite narrow in comparison of its plasma levels between effective analgesia and significant respiratory depression (Yassen et al., 2006; Yassen et al., 2007; Yassen et al., 2008; Boom et al., 2013), By contrast, NSAIDS would be an ideal option of treatment for tonsillectomy pain with a lower incidence of PONV and non-detrimental impact on respiration, but the concern over the potential postoperative hemorrhage caused by NSAIDS has limited its clinical application (Marret et al., 2003; Møiniche et al., 2003; Lake and Khater, 2004). Nalbuphine is a 6-transmembrane MOR agonist as is buprenorphine and butorphanol all of which have a ceiling on respiratory depression (Davis et al., 2018). In addition, Nalbuphine is an opioid agonist–antagonist, active on mu and kappa receptors (Martinelli et al., 2014) to provide analgesia and certain anti-pruritic effects (Romagnoli and Keats, 1980) and have less undesirable outcomes. It has been shown to be safe and effective when used in variety of surgical procedures. The aims of our study were to assess and compare nalbuphine to fentanyl regarding their pain-relief efficiency and the impact on respiration in pediatric patients underwent adenotonsillectomy.

Studies have shown that the ratio of potency between nalbuphine and fentanyl is about 1:100 on a milligram basis, the same as the ratio between morphine and fentanyl. Therefore, we used the equipotent dose of 0.1 mg/kg nalbuphine vs. 1 µg/kg fentanyl as the initial analgesics separately in our study groups (Beaver and Feise, 1978; Twycross, 2007).

Our study showed that patients in nalbuphine group had overall relatively lower average of FLACC scores during the entire course of PACU than ones in fentanyl group, and the statistical significance for the difference can be seen particularly at the time of arriving PACU (0 min). The total numbers of administered rescue analgesics were 80 vs. 156 when comparing nalbuphine and fentanyl groups. Those results indicated that nalbuphine provided a better postoperative pain-relief effect than fentanyl in children undergoing adenotonsillectomy. Hari Prasad et al. compared these two drugs in other surgical patients under general anesthesia and he found that the time to first rescue dose analgesic top up was significantly longer in nalbuphine group than in fentanyl group. They concluded that nalbuphine provides excellent postoperative analgesia which is comparable to fentanyl at a less frequent dosing thus decreasing the overall opioid requirement (Prasad et al., 2016). By acting on μ1 and μ2 receptors, fentanyl is a stronger agonist than nalbuphine to produce analgesia and respiratory depression, and by acting on kappa receptors, nalbuphine may provide better effects of analgesia and sedation (Chen et al., 1993). Specifically, nalbuphine acts as a moderate-efficacy partial agonist or antagonist of the µ-opioid receptor (MOR) and as a high-efficacy partial agonist of the κ-opioid receptor (KOR) (Schmidt et al., 1985; Chen et al., 1993), which has the potential to maintain or even enhance μ-opioid-based analgesia while simultaneously mitigating the µ-opioid side effects (Prabhakaraiah et al., 2017).

Our results revealed that patients in both group had comparably satisfactory pain control in the first 45 min or so after PACU arrival and then, there were significantly increased case numbers with FLACC scores ≥4 in fentanyl group at 1 and 2 h time points, which is consistent with N. Rawal’s study results (Rawal and Wennhager, 1990). A rational explanation for this phenomenon was that the analgesic duration of fentanyl is about 30–60 min and its action began to wear off within 1 or 2 h after patient’s arrival in PACU while nalbuphine’s lasts approximately 3–6 h (slightly longer than morphine) and would provide sufficient pain-relief for the patients during their entire PACU stay and even beyond. The reasons for the increase in number of children who FLACC ≥4 may be multi-factorial.

Nalbuphine has been known to cause respiratory depression by a dose-related ceiling effect (Romagnoli and Keats, 1980) in which, repeated dosing or increased dose of nalbuphine will not further aggregate depressed respiration. Our study showed that none of patients developed hypoxemia and only one had slow RR ≤10/min in nalbuphine group vs. 10 patients developed hypoxemia and 29 had slow RR ≤10/min in fentanyl group. 25 out of 29 patients in fentanyl group developed slow RR within the first 20 min during their PACU stay when fentanyl had reached its peaking effect.

The statistically significant low average SpO_2_ level and slow RR in fentanyl group indicated that fentanyl causes greater respiratory depression than nalbuphine does. The more frequent and marked episodes of hypoxemia observed in the fentanyl group also support this finding. Most likely, this phenomenon is attributed by nalbuphine’s ceiling effect on respiratory depression which will not be worsen further when the dose of nalbuphine has reached a certain threshold. One study has found that nalbuphine, as high as 0.8 mg/kg, will not aggregate respiration depression (Bone and Tooley, 1989).

The main drawback of nalbuphine was the markedly prolonged awaking time postoperatively as reported by some investigators (Vatashsky and Haskel, 1986; Pugh and Drummond, 1987). It is generally believed that nalbuphine is a longer-acting opioid with a half-life of approximately 4 h and it is expected to extend the recovery time significantly. However, we found that the recovery time of nalbuphine group was not significantly different from the fentanyl group.

In summary, nalbuphine provides more effective pain relief than fentanyl in children underwent adenotonsillectomy, which was demonstrated by less intensity of the pain and longer duration of its analgesic effect. Clinical nalbuphine dosage only induces mild respiration depression and does not slow down anesthesia emergence and extubation process, and it will not delay PACU discharge, or increase the incidence of PONV.

### Limitation and Future

Nalbuphine is an agonist/antagonist opioid and is only used as a sole opioid analgesic. And it also has analgesic “ceiling effect” and may not be sufficient to relieve severe pain encountered in other types of surgeries. In this study, we only observed the effect of one fixed dosage of nalbulphine on postoperative pain in children undergoing adenotonsillectomy and future study to find out the optimal dosage(s) for pain management is warranted.

## Data Availability Statement

The raw data supporting the conclusions of this article will be made available by the authors, without undue reservation.

## Ethics Statement

The studies involving human participants were reviewed and approved by Second Affiliated Hospital and Yuying Children’s Hospital, Wenzhou Medical University. The patients/participants provided their written informed consent to participate in this study.

## Author Contributions

All authors listed have made substantial, direct, and intellectual contribution to the work and approved it for publication.

## Funding

The Special Project for Significant New Drug Research and Development in the Major National Science and Technology Projects of China (2020ZX09201002) and Clinical Research Foundation of the Second Affiliated Hospital of Wenzhou Medical University (SAHoWMU-CR2018-03-126).

## Conflict of Interest

The authors declare that the research was conducted in the absence of any commercial or financial relationships that could be construed as a potential conflict of interest.
