# Supplementary figures and images for: Comparison of Postoperative Analgesic Effects Between Nalbuphine and Fentanyl in Children Undergoing Adenotonsillectomy: A Prospective, Randomized, Double-Blind, Multicenter Study
Source: Front Pharmacol. 2020 Dec 9;11:597550. doi: 10.3389/fphar.2020.597550 (PMC7849154; doi:10.3389/fphar.2020.597550)

A

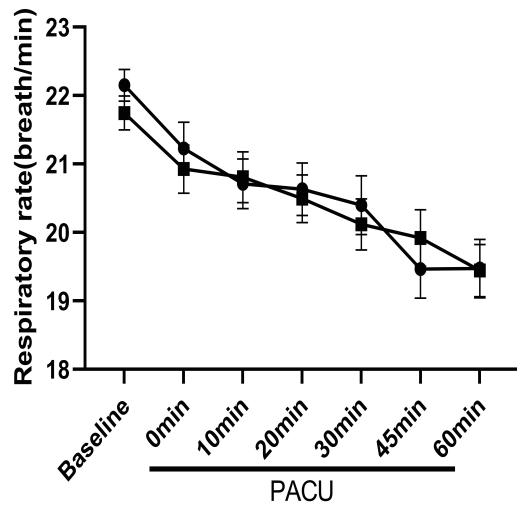

B

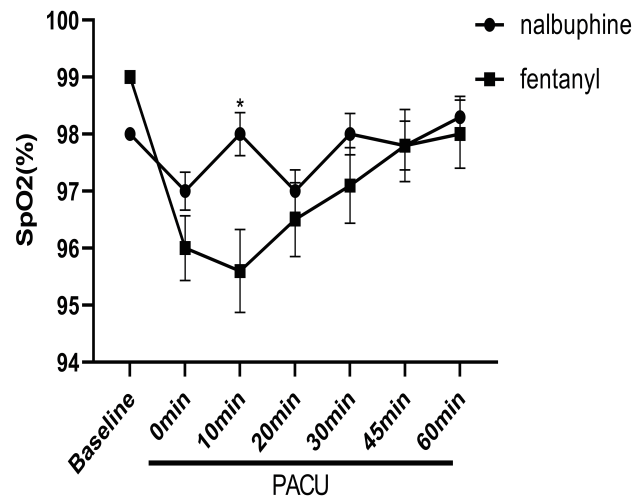

C

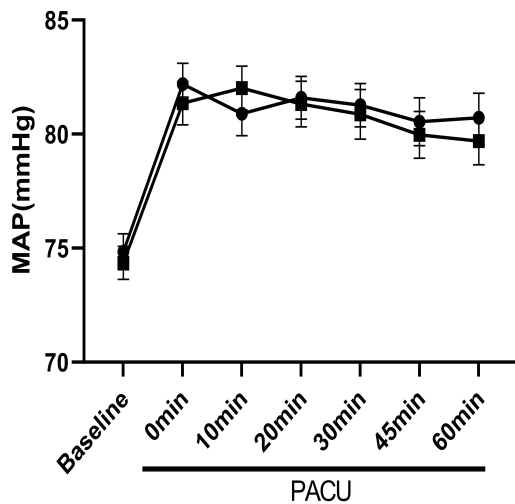

D

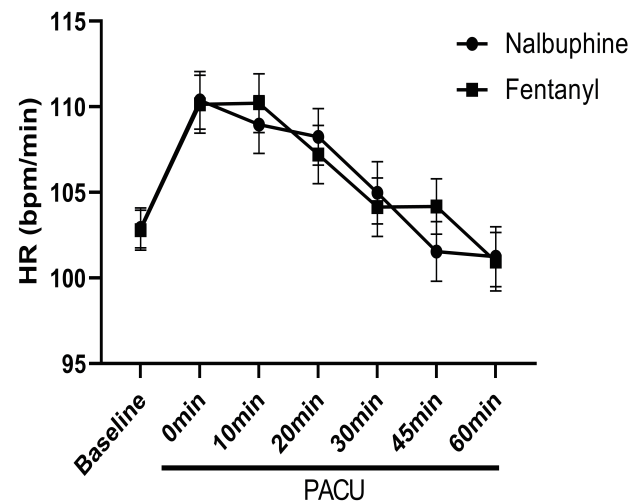

Supplement: Supplementary file 1 [file image1.pdf]
