# Supplementary material for: Comparison of Postoperative Analgesic Effects Between Nalbuphine and Fentanyl in Children Undergoing Adenotonsillectomy: A Prospective, Randomized, Double-Blind, Multicenter Study
Source: Front Pharmacol. 2020 Dec 9;11:597550. doi: 10.3389/fphar.2020.597550 (PMC7849154; doi:10.3389/fphar.2020.597550)
Supplement: Supplementary file 2 [file presentation1.pptx]

## Slide 1
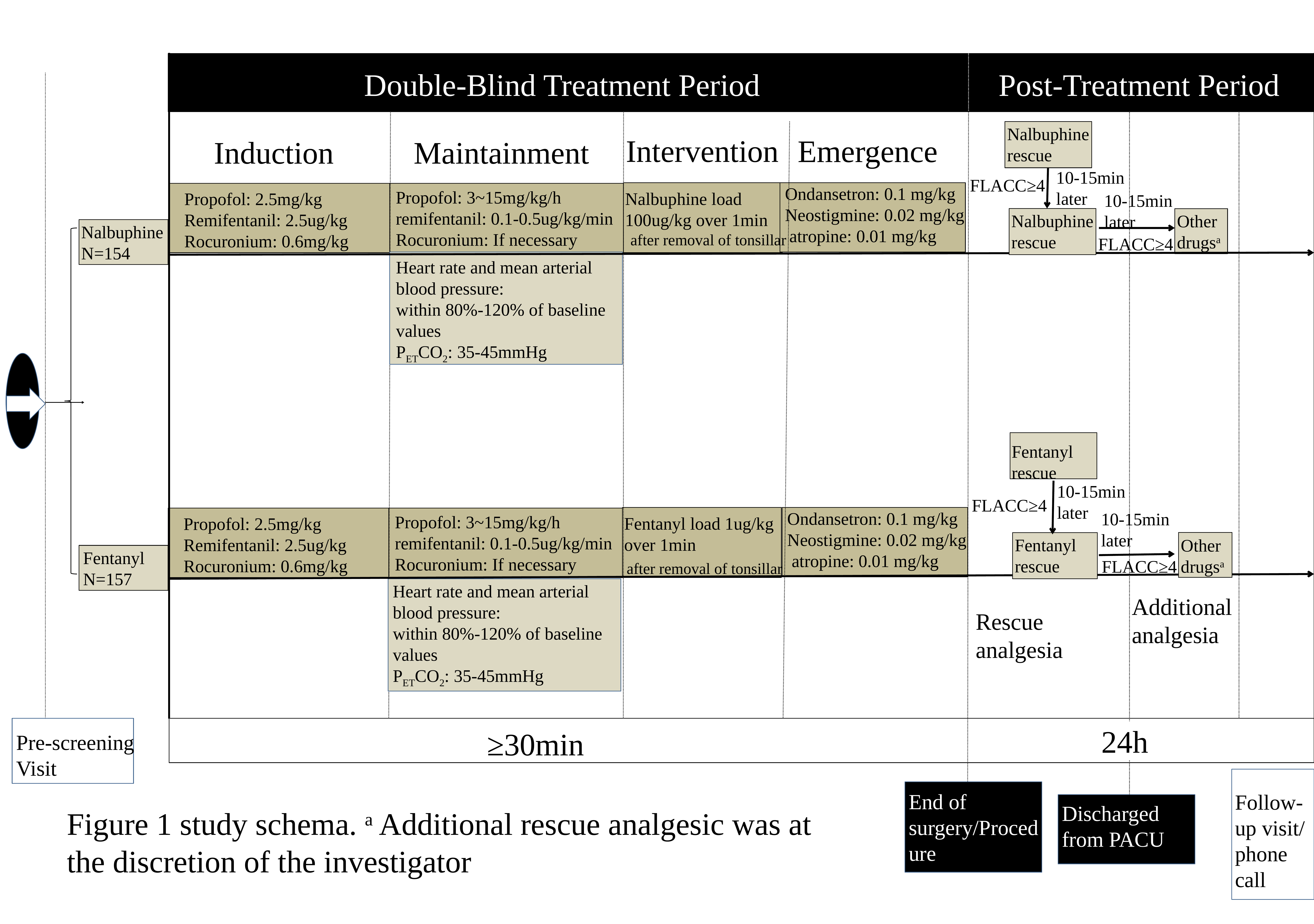

Double-Blind Treatment Period
Post-Treatment Period
Intervention
Nalbuphine rescue
Emergence
Induction
Maintainment
10-15min later
FLACC≥4
Ondansetron: 0.1 mg/kg
Neostigmine: 0.02 mg/kg
 atropine: 0.01 mg/kg
Nalbuphine load 100ug/kg over 1min
Propofol: 2.5mg/kg
Remifentanil: 2.5ug/kg
Rocuronium: 0.6mg/kg
Propofol: 3~15mg/kg/h
remifentanil: 0.1-0.5ug/kg/min
Rocuronium: If necessary
10-15min later
Nalbuphine rescue
Other
drugsa
Nalbuphine
N=154
after removal of tonsillar
FLACC≥4
Heart rate and mean arterial blood pressure:
within 80%-120% of baseline values
PETCO2: 35-45mmHg
Fentanyl rescue
10-15min later
FLACC≥4
Ondansetron: 0.1 mg/kg
Neostigmine: 0.02 mg/kg
 atropine: 0.01 mg/kg
10-15min later
Fentanyl load 1ug/kg over 1min
Propofol: 2.5mg/kg
Remifentanil: 2.5ug/kg
Rocuronium: 0.6mg/kg
Propofol: 3~15mg/kg/h
remifentanil: 0.1-0.5ug/kg/min
Rocuronium: If necessary
Other
drugsa
Fentanyl rescue
Fentanyl
N=157
FLACC≥4
after removal of tonsillar
Heart rate and mean arterial blood pressure:
within 80%-120% of baseline values
PETCO2: 35-45mmHg
Additional analgesia
Rescue analgesia
Pre-screening Visit
24h
≥30min
Follow-up visit/ phone call
End of surgery/Procedure
Discharged from PACU
Figure 1 study schema. a Additional rescue analgesic was at the discretion of the investigator
