# Supplementary material for: Comparison of Postoperative Analgesic Effects Between Nalbuphine and Fentanyl in Children Undergoing Adenotonsillectomy: A Prospective, Randomized, Double-Blind, Multicenter Study
Source: Front Pharmacol. 2020 Dec 9;11:597550. doi: 10.3389/fphar.2020.597550 (PMC7849154; doi:10.3389/fphar.2020.597550)
Supplement: Supplementary file 3 [file presentation2.pptx]

## Slide 1
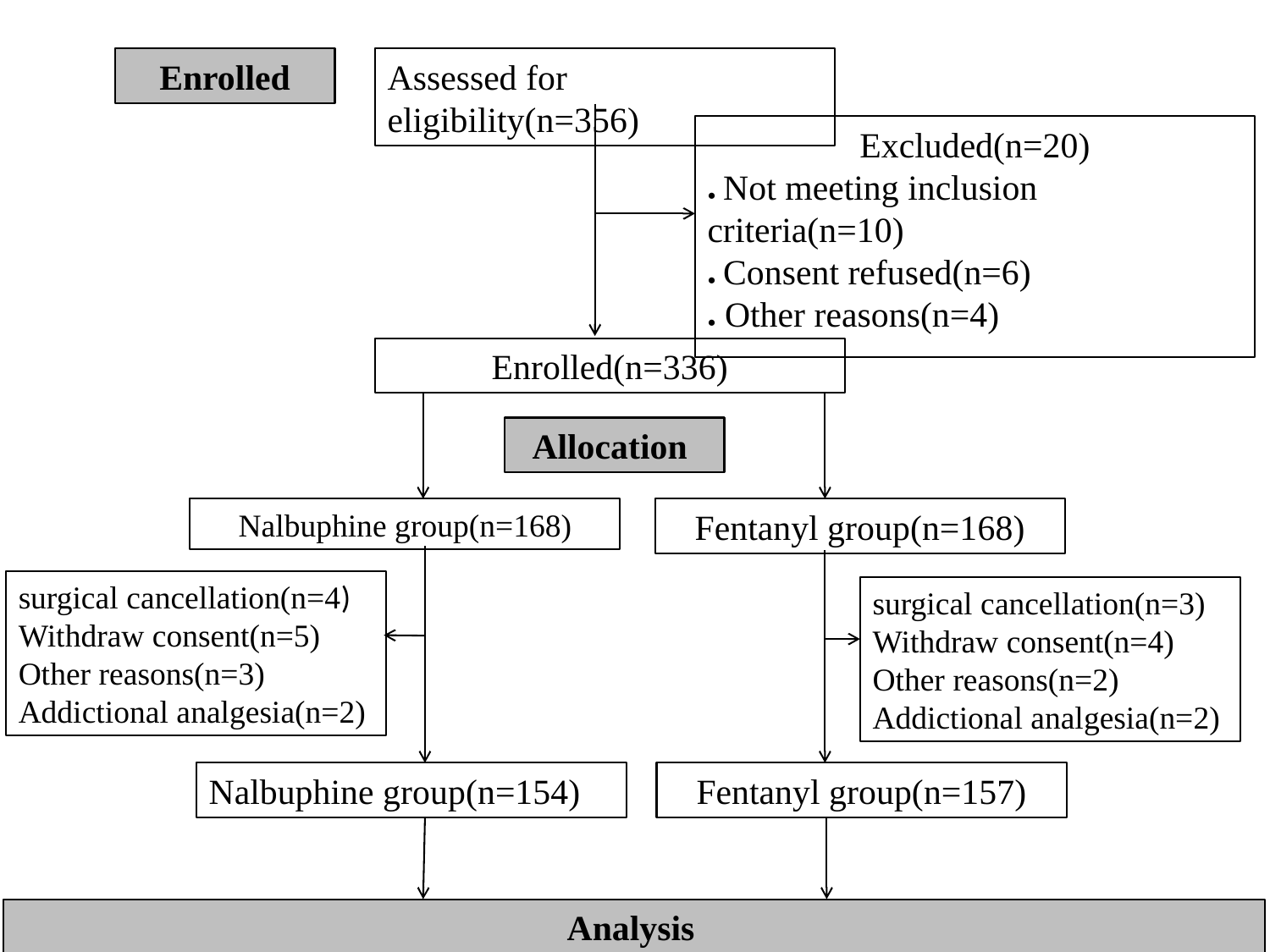

Enrolled
Assessed for eligibility(n=356)
Excluded(n=20)
● Not meeting inclusion criteria(n=10)
● Consent refused(n=6)
● Other reasons(n=4)
Enrolled(n=336)
Allocation
Nalbuphine group(n=168)
Fentanyl group(n=168)
surgical cancellation(n=4)
Withdraw consent(n=5)
Other reasons(n=3)
Addictional analgesia(n=2)
surgical cancellation(n=3)
Withdraw consent(n=4)
Other reasons(n=2)
Addictional analgesia(n=2)
Nalbuphine group(n=154)
Fentanyl group(n=157)
Analysis
